# Supplementary material for: BdNub Is Essential for Maintaining gut Immunity and Microbiome Homeostasis in Bactrocera dorsalis
Source: Insects. 2023 Feb 10;14(2):178. doi: 10.3390/insects14020178 (PMC9964267; doi:10.3390/insects14020178)
Supplement: Supplementary file 1 [file insects-14-00178-s001.zip › insects-2127451-supplementary.pdf]

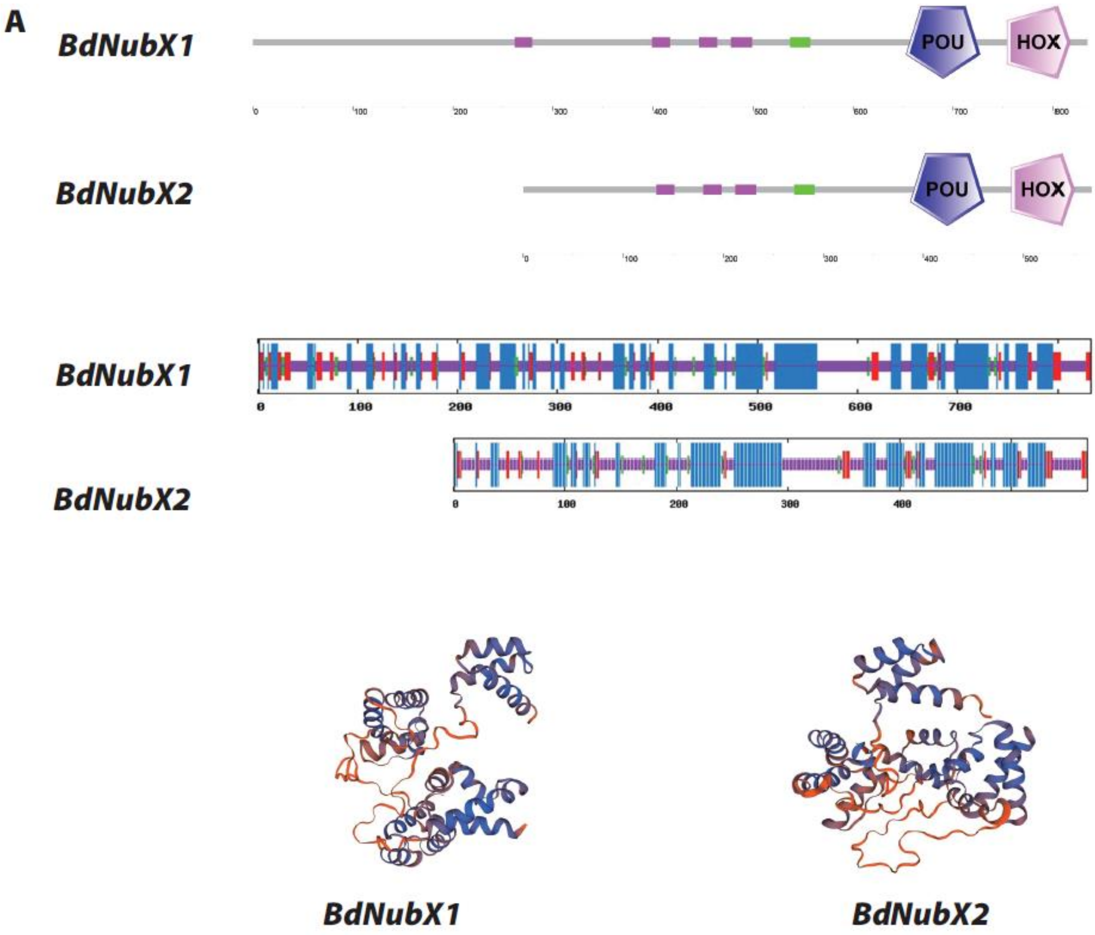

FigureS1. Protein functional domain analysis of *BdNubX1* and *BdNubX2*. A:From top to bottom, Protein function domain analysis of *BdNubX1* and *BdNubX2*. Prediction the of secondary structure of *BdNubX1* and *BdNubX2*. Prediction of the tertiary structure of *BdNubX1* and *BdNubX2*
